# Psychological and Spiritual Support for Parents of a Premature Baby in the Intensive Care Unit: Scoping Review

**DOI:** 10.3390/healthcare13192478

**Published:** 2025-09-29

**Authors:** Barbara Kegl, Urška Novak, Rosemarie Franc, Nataša Mlinar Reljić

**Affiliations:** 1 Faculty of Health Sciences, University of Maribor, Žitna ulica 15, 2000 Maribor, Slovenia; barbara.kegl@um.si (B.K.); urska.novak4@student.um.si (U.N.); 2 University Division of Gynaecology and Perinatology, University Medical Centre Maribor, Ljubljanska ulica 5, 2000 Maribor, Slovenia; rosemarie.franc@ukc-mb.si

**Keywords:** parents, preterm infant, intensive care unit, psychological support, spiritual care

## Abstract

**Background:** Psychological and spiritual support is crucial, especially in challenging life situations, trials, and when facing the unimaginable distress experienced by parents of premature babies in the intensive care unit. This scoping review aims to identify the psychological and spiritual support needed by parents of premature babies in intensive care units. **Methods:** The databases PubMed, CINAHL Ultimate and Web of Science were searched in April 2025. The Preferred Reporting Items for Systematic Reviews and Meta-Analyses extension for Scoping Reviews guidelines were followed. The extraction table was used to extract significant information from each source of evidence. Descriptive data analysis was used to present results. **Results:** The search identified 1353 hits, and 17 sources of evidence were included in the review. The results indicate that psychological and spiritual support has been implemented in clinical practice. The main category Psychological and spiritual needs of parents of preterm babies in intensive care was designed using subcategories: psychological needs of parents of preterm babies in intensive care, and spiritual needs of parents of preterm babies in intensive care. **Conclusions:** In the context of continuous and holistic healthcare for preterm babies in intensive care units, psychological and spiritual support represent essential and interrelated components. Spiritual support for parents is frequently underrecognized and insufficiently addressed.

## 1. Introduction

Premature birth, defined as delivery before 37 weeks of gestation, occurs when fetal organs are not yet fully developed to sustain independent life outside the womb [1]. Globally, preterm birth remains a major public health concern, with approximately 13.4 million babies born prematurely in 2020 alone [2]. Rates of preterm birth vary significantly across countries, ranging from 4% to 16% of all live births [2]. In Slovenia, data from 2022 showed that out of 17,381 live births, 1038 were preterm [3]. While the birth of a child is typically a joyful event, premature delivery often induces fear, anxiety, and uncertainty for parents, disrupting their expectations and psychological well-being [4,5,6,7].

Parents of preterm infants are at heightened risk for psychological distress, including anxiety, depression, and post-traumatic stress disorder [8]. The need for intensive care exacerbates this vulnerability by intensifying emotional stress and contributing to spiritual struggles [9]. Brelsford et al. [9] found that higher levels of neonatal intensive care unit (NICU) related stress were significantly associated with increased psychological and spiritual distress, along with reduced cognitive well-being. A meta-analysis by Yildiz et al. [8] further revealed that 18.5% of mothers of preterm or critically ill infants met diagnostic criteria for post-traumatic stress disorder, underscoring the severity of mental health challenges in this population.

Emotional and spiritual support are crucial for helping parents cope with the complexities of premature birth. Family-centred care approaches, skin-to-skin contact, parental education programmes, interpersonal psychotherapy, and spiritual care have shown beneficial effects on parental well-being and parent–infant bonding [10]. Nurses and midwives play a key role in facilitating these interventions, as they often serve as the primary point of contact and support for families during NICU hospitalization [6,10]. Additionally, guiding parents toward peer support, social media groups, and emotional support networks can foster resilience and reduce isolation [10]. Spiritual self-care practices have been shown to reduce stress and anxiety among mothers of premature infants, providing a valuable nonpharmacological complement to psychological support [11]. In this review, psychological support is understood as general emotional or psychosocial support provided by nurses and midwives. Professional psychotherapy and counselling require specialized training and are explicitly distinct from nursing and midwifery roles. But, in NICU practice, nurses and midwives are often the first point of contact for parents and provide day-to-day emotional support, empathetic, compassionate communication, and reassurance, which we classify as psychological support.

This study adopts Watson’s Theory of Human Caring [12] as its guiding framework for understanding and providing spiritual care to premature babies’ families in the NICU. Watson’s theory views nursing as a transpersonal process in which the nurse engages with the whole person—body, mind and spirit—through authentic presence, intentionality, and the creation of healing environments [13]. Central to the theory are the caritas processes, which emphasize loving-kindness, faith, hope, cultivation of spiritual practices, deep listening, and sustaining human dignity even in highly technical settings [12] like the NICU. In the NICU context, this approach invites nurses to move beyond task-oriented care to include spiritual assessment, compassionate communication, support for parental meaning-making, and culturally appropriate rituals, all of which may buffer distress and strengthen psychological family–infant bonds [14].

In this review, spirituality is understood as the dynamic dimension of human life that relates to the way persons experience, express and seek meaning and purpose, and the way they connect to the moment, to self, to others, to nature, to the significant or the sacred [15]. This definition highlights that spirituality is broader than religion, though religious traditions and practices may be one way in which spirituality is expressed. Psychological support is closely connected to spirituality, as mental health, emotional well-being, and coping cannot be fully separated from questions of meaning, purpose, and hope [16]. For this reason, psychological and spiritual support should be viewed as interrelated dimensions of nursing and midwifery care. Psychological support primarily addresses emotional well-being and mental health, while spiritual support encompasses meaning-making, purpose, connecting, both religious and non-religious ways of coping, and sustaining resilience in the context of parents of premature babies in NICU.

Despite growing awareness of the psychological and spiritual burden faced by NICU parents, research remains fragmented, and comprehensive, integrative care models are lacking. Current literature also highlights disparities in how mothers and fathers experience and express distress, pointing to the need for tailored, inclusive support strategies [5,7,9].

This scoping review aims to map and synthesize the current evidence on the psychological and spiritual needs of parents of preterm infants in the NICU. The review underscores the importance of integrating psychological and spiritual support into nursing care in NICU practice to promote parental resilience, emotional well-being, and positive long-term outcomes for families.

## 2. Materials and Methods

### 2.1. Design

This study used a scoping literature review methodology guided by the Joanna Briggs Institute (JBI) Scoping Review Methodology, with nine steps [17]. It is used as a scoping review that systematically explores and maps the existing literature on a topic, providing a broad and comprehensive overview of available research [17]. The Preferred Reporting Items for Systematic Review and Meta-Analyses extension for Scoping Reviews (PRISMA-ScR) guidelines were conducted [12,13]. A scoping review protocol has been registered through OSF Registries (https://doi.org/10.17605/OSF.IO/FYVPC) as recommended by Pollock et al. [18].

### 2.2. Eligibility Criteria

The Population, Concept, Context (PCC) approach was used for developing a research question as recommended by Peters et al. [17], which leads to an efficient search for information and knowledge regarding psychological and spiritual support for parents with preterm babies in the NICU. The research question was “What psychological and/or spiritual support (C) do parents of a preterm baby (P) need in the intensive care unit (C)?”

The eligibility criteria (Table 1) followed the PCC framework [17]. Sources based on qualitative, quantitative methodological design, mixed-method studies and review articles related to psychological and spiritual support of parents with preterm babies in the NICU were included, with the limitation of English language. Following the recommendations to include grey literature in scoping reviews [17], all sources of scientific literature related to psychological and spiritual support for parents of premature babies were included. Non-empirical evidence, like policy papers, editorials, books and discussion papers, was excluded from this scoping review.

### 2.3. Search Strategy

The databases PubMed, Cumulative Index to Nursing and Allied Health Literature (CINAHL Ultimate) and Web of Science were systematically searched in April 2025 with search strategy developed using keywords and synonyms combined with the Boolean operators AND and OR as follows: ((“emotional support” OR emotional* OR feeling* OR spiritual* OR “spiritual care” OR “spiritual support” OR “spiritual need”) AND (“NICU” OR “neonatal intensive care unit” OR “newborn intensive unit” OR “intensive care”) AND (parents OR mother OR father OR family)). The search strategy was developed using the PCC framework and validated through a pilot search in PubMed. Key articles known to the research team were used as reference points to ensure sensitivity, and the number of irrelevant hits was assessed to maintain specificity. The final list of keywords and Boolean combinations was agreed upon by all authors before applying the strategy to PubMed, CINAHL Ultimate, and Web of Science.

The final hits were uploaded to the Mendeley Reference Management Program to remove duplicates. In the next step, the titles and abstracts were screened. Three authors (UN, NMR, BK) independently screened titles and abstracts and resolved disagreements in discussion with the coauthor (RF). In the next step, all sources were read as full texts and included in the final analysis if they met all inclusion criteria and the agreement of the entire author team.

### 2.4. Data Extraction and Analysis

Data extraction was performed independently by three reviewers using a standardized data extraction form [19] that had been pilot-tested on a small number of studies to ensure clarity and consistency. The form included details such as study design, setting, participants, and key findings related to psychological and spiritual support. It was adjusted as new information emerged during the review process, reflecting the iterative nature of data extraction in scoping reviews [20]. Disagreements between reviewers were discussed until consensus was reached, with a fourth reviewer consulted when necessary. Descriptive data analysis was used, as scoping reviews are exploratory and aim to map the range of existing evidence and provide summaries, not synthesizing findings [20]. A descriptive qualitative approach involves basic data coding and organizing it into categories, focusing on concepts relevant to the research question [19]. Although no formal inter-rater reliability statistics were calculated, the use of independent extraction by three reviewers and consensus discussion helped ensure accuracy and reduce potential bias.

## 3. Results

The survey investigates scientific literature concerning psychological and spiritual support for parents of premature babies within NICUs.

**Figure 1:**
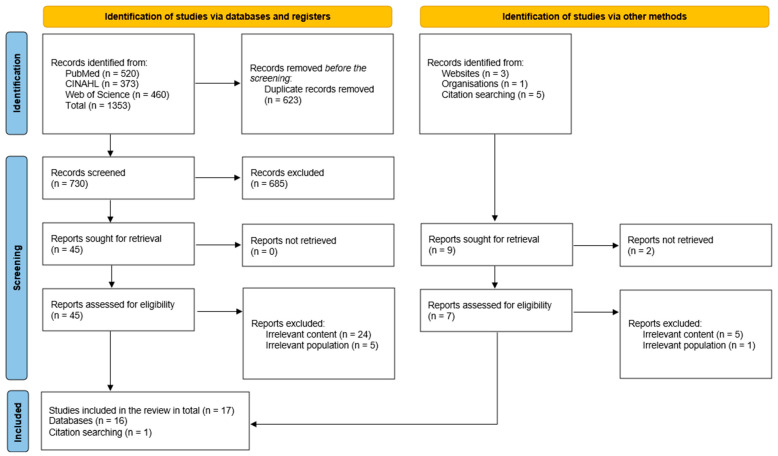
PRISMA flow diagram of the search process [36].

In total, 1353 results were identified from the following databases: CINAHL Ultimate (*n* = 373), PubMed (*n* = 520), Web of Science (*n* = 460) (see Supplementary Table S1). After removing duplicates (*n* = 623), the results were screened by title and abstract (*n* = 730). Based on the title and/or abstract, results (*n* = 685) were excluded as they did not meet the inclusion criteria. The process proceeded with a full review of articles (*n* = 45). Based on exclusion criteria, we excluded articles due to irrelevant content (*n* = 24) and irrelevant population (*n* = 5). One study for final analysis was included from citation searching. Seventeen studies met the inclusion criteria and were synthesized for analysis as presented in Figure 1.

Seventeen studies were included in the final analysis. Four studies were conducted in Italy [5,21,22,23], three in the United Kingdom [24,25,26]. Two studies were conducted in Turkey [27,28], Spain [29,30], and Iran [10,31], one in Israel [32], one in Australia [33], Portugal [34] and Norway [35]. The total sample included in the studies consisted of parents (*n* = 2261) and healthcare workers (*n* = 157). The included studies, as presented in Table 2, vary in methodology and are categorized according to the authors’ methodological descriptions as follows: nine studies based on quantitative [5,21,22,27,28,30,32,33,35], five studies based on a qualitative methodological design [24,25,26,29,31]. Two studies used mixed methods [23,34], and one study was a review [10].

The descriptive analysis yielded the following one main category: Psychological and spiritual needs of parents of preterm babies in the NICU and two subcategories: (a) Psychological needs of parents of preterm babies in the NICU and (b) Spiritual needs of parents of preterm babies in the NICU (Table 3).

The first subcategory describes the psychological needs of parents of premature babies in the NICU. Parents of premature babies often experience intense emotional distress, including sadness, anxiety, guilt, and despair [29,35], especially in the first week after birth [10,29]. Separation from family worsens these feelings [29,35], and many parents express a strong need to discuss their feelings and emotions [10,29]. They also face existential concerns about their infant’s survival and health [26,34], emphasizing the need for accessible and supportive healthcare professionals [21,25]. Mothers process emotions differently from fathers [21,33]. Fathers tend to detach emotionally and focus on technical details [21], while mothers experience higher postpartum distress, particularly after preterm birth. Poor maternal mental health is directly linked to the neurological development of infants born at ≤32 weeks, highlighting the importance of psychological support [27]. Healthcare professionals play a key role in recognizing and addressing emotional needs [24,26,31,34]. Parents expect availability, empathy, and compassion, as emotional support is essential for stress prevention and overall well-being [22,23,26,33]. Single-room accommodations with preterm infants improve parental psychological outcomes, reinforcing the need for holistic, family-centred neonatal care [35].

The second subcategory describes the spiritual needs of parents of premature babies in the neonatal ICU. Parents of preterm babies often seek spiritual support to cope with distress, as spirituality is closely linked to psychological well-being [10,28,31]. The need for hope is particularly strong [24], leading many parents to turn to religious texts such as the Bible or the Quran for comfort and reassurance [24]. Spirituality plays a crucial role in times of crisis, with parents incorporating cultural and religious elements into their coping strategies. Many parents bring protective amulets, such as the evil eye, red bells, the hamsa (Fatima’s hand), Christian crosses, and rosaries, believing these will safeguard their preterm baby [30]. Some also carry amulets for personal protection and occasionally hide them from healthcare providers [31]. Recognizing this deep connection to faith, some NICU have introduced religious imagery to support parents spiritually [28]. Parents frequently seek information about their infant’s condition, yet nurses often redirect them to doctors due to time constraints [31]. The way parents engage with spirituality is shaped by their personal relationships, cultural norms, and coping mechanisms, particularly among fathers [31]. Additionally, spiritual care has been shown to reduce maternal stress levels, highlighting its importance in NICU [31].

## 4. Discussion

This scoping review mapped the existing literature on psychological and spiritual support for parents of preterm infants during NICU hospitalization. The applicability of our findings must be interpreted in the context of cultural and organizational diversity across NICU. The studies included in this review originated from Europe, the Middle East, and Australia, reflecting variations in family structures, spiritual traditions, and psychological expectations surrounding parenthood. Cultural norms shaped how mothers and fathers expressed distress, sought support, and engaged with psychological and spiritual aspects. Healthcare systems also influenced the scope and delivery of support, including the availability of single-family rooms, integration of chaplains, advanced nurses in spiritual care, psychologists in NICU teams, staffing levels, and policies regarding parental presence. For example, in high-income countries, strategies often focus on structural and organizational resources, such as single-family rooms, integration of psychologists and chaplains into NICU teams, and digital peer-support platforms. In contrast, in low- and middle-income countries, where such resources may be limited, emphasis may need to be placed on strengthening basic emotional support from nurses and midwives, facilitating peer-to-peer parent groups, incorporating cultural and religious coping practices, and providing low-cost training in therapeutic communication. These differences suggest that interventions should be context-specific, adapted to available resources and aligned with local cultural values. These contextual factors suggest that strategies for psychological and spiritual care cannot be assumed to transfer seamlessly between settings; rather, they should be adapted to local cultural values, family expectations, and institutional resources.

Parents of preterm babies often experience intense psychological distress, particularly in the early postpartum period. Emotional responses such as stress, anxiety, helplessness, and depression are common, especially within the first week following birth. It is important to acknowledge that mothers and fathers often process the NICU experience differently. Fathers often exhibit emotional detachment or focus on technical details, while mothers report higher levels of postpartum distress. Given the critical influence of maternal well-being on neonatal neurological development, targeted psychological support for mothers is essential. Nevertheless, fathers also often experience anxiety and reduced quality of life, necessitating mental health screening and support tailored to their unique coping styles. First-time parents face even greater psychological challenges as they simultaneously adjust to parenthood and navigate the complexities of neonatal care. Some studies [37] also report that maternal mental health, in particular, plays a pivotal role in preterm babies’ outcomes as high levels of maternal stress are associated with adverse neonatal development, especially in infants born before 32 weeks of gestation. So, it is necessary to provide family-centred interventions, including skin-to-skin care in NICUs with active parental involvement in caregiving, which have been shown to bolster parental confidence and enhance emotional well-being [10]. Although psychological distress was consistently documented in the included studies, relatively few [25,26,34] described structured psychological interventions delivered by trained professionals such as psychologists or psychotherapists. Most psychological contributions reported in the literature were embedded within nursing- and midwifery-led approaches, including family-centred care, peer support, and parental education, rather than formal psychotherapy. This imbalance suggests that while parents’ psychological needs are widely recognized, systematic implementation of professional psychological interventions in NICUs remains limited. Addressing this gap is an important direction for future research and practice, particularly in developing integrative models where professional psychological care complements the supportive roles of nurses and midwives. It is important to note that most of the psychological support described in the included studies did not involve structured therapy by psychologists, but rather psychosocial and emotional support delivered by nurses and midwives. This included active listening, empathetic presence, family-centred communication, and facilitating peer connections. While these do not replace professional psychological therapy, they represent essential frontline strategies within holistic nursing and midwifery care, especially in settings where psychologists are not consistently available as part of NICU teams.

Spirituality emerges as a significant coping mechanism for many parents during the neonatal ICU experience. Parents often draw strength from religious texts, symbols, or amulets, while some nurses and midwives integrate spiritual elements into the ICU environment to address these needs. Providing spiritual care in the NICU improves parents’ emotional stability and coping capacity. Spiritual support reduces stress and enhances resilience, particularly in culturally diverse settings [14]. The psychological and spiritual assessment of parents’ needs is necessary in the NICU to understand parents’ needs and to prepare a family-centred nursing care plan. This can enhance emotional stability and foster stronger parent–preterm infant bonding [38].

Effective communication between healthcare professionals and parents is another cornerstone of psychosocial support. While parents often seek detailed information about their preterm’s condition, nurses and midwives may be constrained by time and workload. Structured communication strategies, such as multidisciplinary family meetings and individualized counselling, have been shown to reduce anxiety and increase parental satisfaction with care [39]. Open dialogue, empathy, and consistent parental involvement also significantly enhance emotional outcomes [26]. Moreover, higher parental participation in NICU care correlates with reduced maternal feelings of helplessness and improved well-being [40]. The implementation of single-family room NICUs has also been linked to lower parents’ emotional distress and maternal depression, strengthened parent–preterm bonding, and improved breastfeeding rates [39]. Peer support initiatives within the NICU further contribute to emotional relief. Programmes that facilitate connections between parents, through in-person groups or digital platforms, help reduce stress and build community, which is consistent with Franck et al. [38] findings.

While psychological and spiritual needs were treated as distinct in this review, they often overlap in practice. Parents may voice psychological distress—such as fear or guilt—through spiritual language, or they may seek reassurance about their baby’s outcome in terms of faith or divine will. At the same time, some concerns reflect a specifically transcendent search for meaning or connection. Nurses and midwives can support families by exploring the source of these concerns and discerning whether they call for counselling, spiritual care, or an integrated response. Integrating psychological and spiritual support into neonatal ICU care is essential for promoting health and well-being. Future research should prioritize the development of practical tools, training programmes, and standardized screening protocols to support holistic, family-centred care in NICU. Expanding access to psychotherapy, fostering a culture of empathy, compassion, caring, active listening, and enhancing therapeutic communication are key strategies for improving outcomes. Additionally, innovations such as parent mentoring programmes and online peer support networks offer promising avenues for providing continuous emotional support [38]. Emotional distress may be alleviated through both psychological counselling and spiritual reassurance, especially when care is tailored to individual parental needs, cultural contexts, and coping styles. Supporting nursing and midwifery staff through training, debriefing, and spiritual and psychological care services enhances their capacity to support parents and sustain a compassionate therapeutic environment.

The findings of this review further reinforce that psychological and spiritual support, while conceptually distinct, are deeply interconnected in practice. Parents’ emotional well-being and coping cannot be separated from their search for meaning, hope, and purpose, as psychological distress is often mediated or expressed through spiritual resources. For example, spiritual coping strategies such as prayer, reliance on faith traditions, or the use of symbolic objects often served not only to meet spiritual needs but also to alleviate anxiety, reduce stress, and enhance resilience. Conversely, targeted psychological support, including counselling or peer support, frequently facilitated parents’ capacity for meaning-making and spiritual growth. These overlapping pathways suggest that psychological and spiritual care should not be approached in isolation but integrated within holistic, family-centred nursing and midwifery practice in the NICU.

The evidence from this review highlights the necessity of addressing both psychological and spiritual dimensions of care for parents in NICUs. By implementing evidence-based interventions, nurses and midwives can ensure that families facing the challenges of preterm birth receive the comprehensive support they need to navigate this complex journey with resilience and confidence.

### 4.1. Implications for Nursing and Midwifery Practice, and Future Research

Based on this review, the authors recommend implementing some interventions into clinical practice to strengthen psychological and spiritual support in NICUs. We recommend: (1) implementing routine screening of both parents for anxiety, depression, and spiritual distress at admission and at set intervals; (2) establishing structured care pathways that combine counselling, peer-support groups, and access to trained chaplains or advance nurse/midwifery spiritual care provider; (3) training nurses and midwives in empathic, compassionate communication, culturally sensitive spiritual care, and early recognition of distress signals; (4) creating parent-friendly environments—such as single-family rooms, private areas for counselling or spiritual practices, and clear information boards to reduce stress and support coping; and (5) introducing parent-mentoring or digital support programmes to provide guidance and connection during and after hospitalization in NICU. These measures are feasible within nursing and midwifery care and can foster resilience, improve parent–infant bonding, and enhance overall outcomes for families facing the challenges of preterm birth.

### 4.2. Limitations

This scoping review has several limitations that should be acknowledged. First, the review included only studies published in English, which may have excluded relevant research published in other languages and limited the cultural diversity of perspectives. Second, although a comprehensive search strategy was employed, it is possible that some relevant studies were missed due to limitations in database indexing or publication bias, especially regarding grey literature and unpublished data. Another limitation is that PsycINFO was not included in the database search. Although this database is an important source for psychological literature, we selected PubMed, CINAHL Ultimate, and Web of Science for their broad coverage of health, nursing, and interdisciplinary studies. While this choice may have excluded some psychology-specific publications, the combination of these databases, together with grey literature searching, provided comprehensive coverage of the field.

Third, although no formal inter-rater reliability statistics were calculated, the use of independent extraction by three reviewers and consensus discussion helped ensure accuracy and reduce potential bias. Fourth, the methodological quality of the included studies was not assessed, in line with the scoping review methodology. As a result, the strength of evidence and risk of bias within individual studies could not be evaluated. Fifth, the heterogeneity of the studies in terms of design, sample size, cultural context, and outcome measures limits the ability to perform direct comparisons. Additionally, the review focused on parental experiences during neonatal ICU hospitalization and did not extend to long-term outcomes or post-discharge needs, which may provide a more comprehensive understanding of psychological and spiritual support over time. Findings should be interpreted with caution as cultural norms and healthcare systems vary across study contexts. Finally, while this review mapped key themes in the literature, it did not explore the perspectives of nurses and midwifery in depth, nor did it examine system-level barriers to implementing psychological and spiritual care in neonatal ICUs. Future research should address these gaps to develop and evaluate targeted interventions that are both culturally sensitive and practically feasible in clinical settings.

As researchers and clinicians, we are deeply connected to the topic of psychological and spiritual support for parents of preterm infants. Our professional roles span nursing and midwifery, and we work with families whose babies are hospitalized in neonatal intensive care units. These encounters have made us aware of the profound emotional and spiritual distress parents often experience, as well as the gaps in systematic support within clinical practice.

Clinical work which is personally touched by prematurity strengthened our motivation to explore how care can be more holistic and compassionate. Undertaking this scoping review allowed us to merge scholarly curiosity with professional commitment, aiming to provide evidence that can guide practice and ultimately enhance parents’ resilience and well-being during one of the most vulnerable times in their lives.

## 5. Conclusions

This scoping review highlights that parents of preterm babies in NICU face profound psychological and spiritual challenges, particularly in the first days after birth. Evidence consistently shows that targeted emotional support, family-centred interventions, and culturally sensitive spiritual care can alleviate distress, foster resilience, and strengthen the parent–infant bond. While psychological and spiritual needs often overlap, both require deliberate, structured attention from healthcare professionals, especially from nurses and midwives. Routine screening, integration of spiritual resources, and training of nurses and midwives in empathic, compassionate communication and culturally competent care should become standard components of neonatal practice.

## Figures and Tables

**Table 1:** Inclusion and exclusion criteria based on the research question.

Research Question	Inclusion	Exclusion
Population	Parents with preterm babies	Studies not involving the parents of preterm babies
Concept	Psychological support and/or spiritual support	Studies without a psychological or spiritual support component
Context	Neonatal Intensive Care Unit	Studies outside NICU settings
Study design	Empirical studies (qualitative, quantitative, mixed-methods, review articles); grey literature relevant to the topic	Non-empirical evidence (policy papers, editorials, discussion papers, books)
Language	English publications	Non-English publications

**Table 2:** Descriptive characteristics of identified studies.

Author, Year, Country	Study Design	Objectives	Sample (*n*)	Main Findings
Amorim et al. (2019), Portugal [34]	Mixed methods, sequential explanatory research design	To explore the needs of parents of premature babies hospitalized in neonatal intensive care units, based on their socioeconomic status, past pregnancies, and the characteristics of the preterm infant.	*n* = 118 mothers and *n* = 98 fathers	Mothers valued the information provided more than fathers. Results were primarily influenced by age and education level, while fathers’ needs were influenced by previous children. Qualitative findings added: need for government support; regular emotional support from psychologists and social workers; improved privacy for providing information and comfort; availability of healthcare workers to provide coherent information.
Assal-Zrike et al. (2021), Israel [32]	Quantitative methodology, cross-sectional design	To understand the emotional distress of Bedouin mothers after giving birth to a preterm infant compared to mothers of full-term newborns.	*n* = 321 mothers, 66 with preterm infants and 255 with full-term infants	Mothers of preterm infants showed higher levels of postpartum emotional distress compared to mothers of full-term infants.
Bozkurt et al. (2016), Turkey [27]	Quantitative methodology, cross-sectional design	To examine the associations between maternal depression and anxiety and neurodevelopmental outcomes of preterm infants with gestational age ≤ 32 weeks, assessed at corrected age of 18 to 22 months.	*n* = 220 preterm infants with gestational age ≤ 32 weeks, hospitalized between January 2008 and September 2011 in a neonatal intensive care unit	Maternal depression was directly and negatively associated with neurodevelopmental outcomes of preterm infants. The psychological well-being of mothers must be considered in long-term follow-up, as it directly affects the neurological development of preterm infants, seen at a later age.
Coppola et al. (2013), Italy [21]	Quantitative methodology, cross-sectional design	To explore the willingness of parents of hospitalized preterm infants to share their experience, the social aspect of sharing, emotions, feelings, perceived benefits, and sources of disagreement.	*n* = 40 parents of preterm infants (20 married couples)	Over 80% of parents felt the need to share the experience, mostly with their partner, within a week of the child’s birth. Fathers’ psychological perspective was mainly related to the infant’s health risk, while mothers’ was tied to their emotional response. Guilt, anger, and lack of communication prolonged coping time.
Heidari et al. (2017), Iran [31]	Qualitative methodology, descriptive qualitative design	To explore factors influencing stress management in Iranian parents in neonatal intensive care units.	*n* = 21 parents of preterm infants	Stress management was most influenced by spirituality, information seeking, hope seeking, maintaining calm, attachment to the preterm infant, and communication with healthcare workers.
Hugill et al. (2013), UK [24]	Qualitative methodology, ethnographic design	To explore fathers’ early experiences with preterm infants in neonatal intensive care units.	*n* = 10 fathers of preterm infants and *n* = 87 healthcare workers employed in neonatal intensive care units	Fathers’ emotional reactions were grouped into emotional withdrawal and control, stereotypes, and mixed feelings. Emotional behaviour stemmed from complex, culturally determined conventions and expectations.
Ionio et al. (2016), Italy [5]	Quantitative methodology, cross-sectional design	To explore maternal and paternal reactions immediately after the birth of a preterm infant in terms of trauma-related symptoms, negative mental states, feelings, and stress associated with the neonatal intensive care unit.	*n* = 21 mothers and *n* = 19 fathers of preterm infants; *n* = 20 mothers and *n* = 20 fathers of full-term infants	Parents of preterm infants experienced preterm birth as a psychologically stressful event, increasing stress, anger, anxiety, and depression. Mothers of preterm infants felt more anxious and depressed than mothers of full-term infants. Both parents of preterm infants expressed more anger than parents of full-term infants.
Küçük Alemdar et al. (2018), Turkey [28]	Quantitative methodology, randomized clinical trial	To explore the effect of spiritual care on stress levels in mothers of preterm infants in neonatal intensive care units.	*n* = 62 mothers	Healthcare workers must recognize and meet the spiritual needs of mothers to alleviate stress. These needs should be identified and addressed appropriately.
Lloreda-Garcia (2017), Spain [30]	Quantitative methodology, cross-sectional design	To emphasize the importance of a holistic approach to families, not just the infants, particularly in the area of spiritual beliefs.	*n* = 93 parents *n* = 70 professionals	Most families expressed religious and spiritual needs during the hospitalization of a preterm infant. Healthcare workers often failed to respond adequately to these needs. Amulets and other ritual objects were more common than expected in Western contexts, symbolizing a need for psychological and spiritual support. Many families believed superstitions were behind their children’s issues.
Maleki et al. (2022), Iran [10]	Systematic review with meta-analysis of randomized clinical trials	To synthesize and integrate current international knowledge on nursing interventions that provide emotional support to mothers of preterm infants in neonatal intensive care units.	*n* = 20 studies	Strategies included family-centred care, kangaroo care, psychological and spiritual support, educational programmes, interpersonal psychotherapy, independent spiritual care, individualized developmental care, quality-of-care evaluation programs, and remote care.
Montirosso et al. (2014), Italy [22]	Quantitative methodology, cross-sectional design	To explore how the quality of developmental care regularly provided in 25 tertiary neonatal intensive care units across Italy affects maternal stress and depression.	*n* = 178 mothers	Findings indicated that multiple interventions beneficial for reducing psychological distress in preterm infants can alleviate parental stress and depressive symptoms.
Petersen & Quinlivan, (2021), Australia [33]	Quantitative methodology, longitudinal study	To track the quality of life and psychological well-being in a cohort of fathers to determine whether preterm birth affected outcomes in these areas.	*n* = 1021 fathers of preterm infants	Anxiety after the birth of a preterm infant was found to affect the quality of life of fathers. Routine screening of fathers can identify those who might benefit from psychotherapy.
Russell et al. (2015), UK [25]	Qualitative methodology, descriptive qualitative study	To explore parents’ views and experiences regarding the care of their preterm infant in neonatal intensive care units.	*n* = 32 mothers and *n* = 7 fathers	Parents were generally satisfied with neonatal intensive care. Three key factors influenced their satisfaction: parental involvement in the infant’s care, breastfeeding challenges, and easy access to their child. Communication and empathy from healthcare workers were crucial to parent satisfaction.
San Rafael-Gutiérrez et al. (2020), Spain [29]	Qualitative methodology, phenomenological design	To analyze the emotional support received by parents of preterm infants admitted to neonatal intensive care units.	*n* = 30 mothers and *n* = 10 fathers	Experiences of preterm birth included sadness, guilt, despair, high stress, anxiety, and uncertainty about the infant’s future. Emotional support from healthcare workers reassured parents that their partner was being cared for. Parents expressed concerns about not being able to have all family members visit. Psychological support from healthcare workers was highly valued but not always available when needed. Informal emotional support from family and the presence of the other partner positively impacted mothers’ guilt.
Stacey et al. (2015), UK [26]	Qualitative methodology, descriptive qualitative design	To better understand the factors that support parents in coping with such a life challenge.	*n* = 9 parents of preterm infants born at 26 weeks or later	Findings revealed that the relationships between healthcare workers and parents are key factors in coping. Most healthcare workers showed empathy and supportive care, but it was not always high-quality. Healthcare workers must feel adequately supported to provide quality care to parents.
Stefana et al. (2021), Italy [23]	Mixed methods, longitudinal study	To explore how fathers experience their role in supporting their partner and their relationship with her during their preterm infant’s stay in neonatal intensive care units.	*n* = 20 fathers of preterm infants hospitalized in neonatal intensive care units	Fathers supporting their partner during the neonatal intensive care unit stay experienced emotional distress and a significant need for psychological and spiritual support, often hidden or suppressed, requiring substantial emotional and physical energy.
Tandberg et al. (2019), Norway [35]	Quantitative methodology, prospective study	To compare emotional distress in the form of depression, anxiety, stress, and attachment outcomes between parents of preterm infants cared for in single-family rooms versus open units.	*n* = 132 parents of preterm infants	Lower depression scores were found in mothers, and lower parental stress was reported in both parents in single-family rooms, confirming that single-family room care contributes to better psychological well-being than open units.

**Table 3:** Descriptive analysis of data.

Main Category	Subcategories	Codes	References
Psychological and spiritual needs of parents of preterm babies in the NICU	Psychological needs of parents of preterm babies in the NICU	Parents verbally express emotional distress. Mostly sadness, anxiety, guilt, and despair, which is present due to separation from the family.	[29,35]
		80% of parents felt a strong need to talk about their emotional distress, which was most intense during the first week after birth	[10,29]
		Parents express existential needs (will the baby survive, will they be healthy). They expect healthcare professionals to be available when providing this information.	[26,34]
		Meeting emotional needs is essential for stress prevention.	[21,23]
		Mothers’ and fathers’ emotional experiences are different.	[21,33]
		Emotional detachment is more common in fathers. They are more interested in the technical details of the child’s condition.	[21]
		Healthcare professionals must recognize various emotional states.	[24,26,31,34]
		Parents expect them to be available when they feel helpless, frightened, or anxious.	[22,26]
		Healthcare professionals have to show empathy and compassion.	[25]
		Improved psychological well-being in single-room accommodation with a preterm infant is crucial.	[35]
		Poor maternal mental health directly affects the neurological development of preterm infants born at ≤32 weeks.	[27]
		Mothers of preterm infants also experience higher postpartum distress compared to those of full-term infants.	[21]
	Spiritual needs of parents of preterm infants in the NICU	Parents express a need for spiritual support.	[31]
		Psychological well-being as spirituality often stems from the mental state.	[10,28]
		Parents of preterm infants have strong needs for hope.	[24]
		Frequently turn to religious texts for comfort, and seek reassurance through spiritual means.	[24]
		Many parents rely on spirituality during times of distress.	[30]
		Incorporating cultural and religious elements such as comfort objects (e.g., soft toys) or protective amulets.	[30]
		In response, healthcare professionals have placed religious imagery within neonatal intensive care units, reflecting the strong connection parents feel toward religion and God.	[28]
		Parents frequently ask questions about their preterm infant’s condition, but nurses often respond by directing them to doctors.	[31]
		Some nurses also report not having enough time to address all parental concerns.	[31]
		Cultural norms significantly shape fathers’ experiences and coping mechanisms in particular.	[31]
		Spiritual care directly impacts maternal stress levels, underscoring its importance in neonatal intensive care settings.	[31]

## Data Availability

No new data were created or analyzed in this study. Data sharing is not applicable to this article.

## Supplementary Materials

The following supporting information can be downloaded at: https://www.mdpi.com/article/10.3390/healthcare13192478/s1. Supplementary Table S1: Number of records identified in each database.
