# Supplementary material for: Psychological and Spiritual Support for Parents of a Premature Baby in the Intensive Care Unit: Scoping Review
Source: Healthcare (Basel). 2025 Sep 29;13(19):2478. doi: 10.3390/healthcare13192478 (PMC12523847; doi:10.3390/healthcare13192478)
Supplement: Supplementary file 1 [file healthcare-13-02478-s001.zip › healthcare-3859045-supplementary.pdf]

Supplementary Table S1. Number of records identified in each database.

Database	Hits retrieved	After duplicates removed	Included for screening
PubMed	520	240	20
CINAHL Ultimate	373	190	15
Web of Science	460	193	10
Total	**1353**	**623**	**45**
